# Quality measurement of out-patient neuropsychological therapy after stroke in Germany: definition of indicators and retrospective pilot study

**DOI:** 10.1186/s12883-021-02092-0

**Published:** 2021-02-17

**Authors:** I. Padberg, S. Heel, P. Thiem, A. Diebel, E. Mordhorst, U. Strohmeyer, A. Meisel

**Affiliations:** 1 Center for Stroke Research, Charité-Universitätsmedizin Berlin, Corporate member of Freie Universität Berlin, Humboldt-Universität zu Berlin, and Berlin Institute of Health, Charitéplatz 1, 10117 Berlin, Germany; 2 Zentrum für ambulante Neuropsychologie und Verhaltenstherapie (Center for out-patient neuropsychology and behavioral therapy), Schleiermacherstraße 24, 10961 Berlin, Germany; 3 P.A.N.P - Praxis für ambulante Neuropsychologie Potsdam (out-patient clinic for neuropsychology Potsdam), Ludwig-Richter-Straße 23, 14467 Potsdam, Germany; 4 Praxis Diebel (out-patient clinic Diebel), Oldenburger Straße 30, 10551 Berlin, Germany; 5 Zentrum für ambulante Rehabilitation (Center for out-patient rehabilitation) ZAR Berlin, Gartenstraße 5, 10115 Berlin, Germany; 6 Praxis für ambulante Neuropsychologie und Psychotherapie (out-patient clinic for neuropsychology and psychotherapy), Wilmersdorfer Str. 125, 10627 Berlin, Germany; 7 NeuroCure Clinical Research Center, Charité-Universitätsmedizin Berlin, Corporate member of Freie Universität Berlin, Humboldt-Universität zu Berlin, and Berlin Institute of Health Charitéplatz 1, 10117 Berlin, Germany; 8 Department of Neurology, Charité-Universitätsmedizin Berlin, Charitéplatz 1, 10117 Berlin, Germany

**Keywords:** Stroke, Out-patient, Quality-indicators, Neuropsychology, Professional-reintegration, Interdisciplinary work, Social work

## Abstract

**Background:**

In contrast to the hospital setting, today little work has been directed to the definition, measurement, and improvement of the quality of out-patient medical and therapeutic care. We developed a set of indicators to measure the quality of out-patient neuropsychological therapy after stroke.

**Methods:**

The indicators cover core and interdisciplinary aspects of out-patient neuropsychological work such as mediation of patients into social care in case of need. Selection of the quality-indicators was done together with a consensus group of out-patient therapists and supported by evidence, validity, reliability as well as estimated relevance and variability with the quality of care. The set of indicators was further tested in a retrospective cohort study. Anonymous data of 104 patients were collected from out-patient clinical records of five clinics between November 2017 and April 2018. Associations between process and outcome quality were estimated exploitatively.

**Results:**

Results allowed for the identification of areas with greater variability in the quality of process care and indicated that attention training as recommended by current guidelines had the lowest overall rate for meeting the quality-aim (met in 44% of the cases). This was followed by time < 1 month until the start of therapy (63% met) and mediation into social care in case of need (65% met). We further observed that overall quality and involving relatives in the therapy was associated with higher rates of professional reintegration (*p*-value = 0.03). However, the need for mediation into social care was associated with a reduced chance for successful professional reintegration (*p*-value = 0.009).

**Conclusion:**

In conclusion, we describe a first set of quality indicators which cover different aspects of out-patient neuropsychological therapy and sufficient variability with care. First data further suggests that meeting the specified quality aims may indeed have relevant effects on outcomes.

**Supplementary Information:**

The online version contains supplementary material available at 10.1186/s12883-021-02092-0.

## Background

Stroke is a major contributor to disability and the second leading cause of death worldwide [1]. An estimated 80% of all stroke survivors experience some form of cognitive impairment during stroke recovery [2]. Systematic data on long-term recovery and cognitive status show a large variability in measures used and domains targeted [2]. Cognitive problems that have been reported frequently during the post-acute phase of stroke (1 year after follow up) include deficits in attention (48.5%), short-term memory (24.5%) and executive functions (18.5%) [3]. Importantly, deficits in these cognitive domains are associated with impairments to perform activities of daily living, which in return have been proven to affect reintegration into work [4]. Due to the potentially beneficial effects of neuropsychological intervention and treatment in this area, the development of systematic tools to study long-term treatment quality and its effects on treatment outcome is important.

Quality indicators can help to monitor whether adherence to evidence-based procedures and treatment guidelines affect long-term treatment outcomes and support the translation of research results into clinical practice. Indicators have been developed for acute stroke care as well as for stroke rehabilitation [5–8] and first data for acute care indicate that adherence to these indicators is associated with relevant long-term outcomes [9]. Today, very few approaches to develop quality indicators for stroke care include long-term follow-ups that also extend to the out-patient aftercare of stroke [10]. Furthermore, aspects concerning the quality of aftercare often come from cohort studies [11]. At the same time, existing quality indicator projects after stroke have been criticized for focusing too much on medical and discarding non-medical aspects of stroke care [12]. Out-patient care differs from the clinical one in many aspects. Due to the fragmented nature of service provision, data collection in the out-patient setting often is highly complex. Furthermore, the provision of comprehensive care is challenged by loss of information at the intersections from in-patient to out-patient care as well as between different out-patient service providers. A major obstacle for the development of quality-indicators for the out-patient setting also is the complexity of post-stroke aftercare ranging from only mildly affected patients to patients with severe motor deficits permanently living in nursing homes. Due to this complexity, it is impossible to develop one set of indicators covering all aspects of stroke aftercare. We decided to focus on neuropsychological therapy as it is directed to the treatment of frequent complications after stroke and affects domains such as cognitive problems and depression. These domains have shown importance in regaining and maintaining social and professional functioning after stroke [4, 13].

Out-patient neuropsychological therapy after stroke involves detailed diagnostics of the potential deficits, functional training, facilitation of compensation and coping mechanisms as well as social skills, help with emotional problems associated with the disease, and involvement of relatives in the therapeutic process. Furthermore, out-patient neuropsychology is directed to support patients in social and professional reintegration and hence often involves interdisciplinary cooperation i.e., with social workers. In Germany, patients are eligible for out-patient neuropsychological therapy if deficits after acquired brain injury relevant to everyday living and working persist after in-patient rehabilitation or become apparent mainly after release from the hospital when the patients are confronted more with problems encountered mainly in the out-patient setting. Psychotherapists who obtained an additional 2-year training in clinical neuropsychology can apply for a license to get reimbursement for out-patient neuropsychological therapy by the national health insurance. Therapists who do not have a general license for reimbursement, however may apply for reimbursement of out-patient neuropsychological therapy on an individual case basis as well.

In the current study, we aimed to develop and test a set of quality indicators for neuropsychological therapy in the out-patient setting using stroke as a model disease for acquired brain injury. To obtain first results on the variability of the general quality of care in out-patient neuropsychological therapy and its relation to long-term outcome, we tested the set of quality indicators in a retrospective pilot study.

To this end, we developed a set of fifteen potential indicators for process quality and one for outcome quality, which can be used as a tool to measure the quality of care in out-patient stroke aftercare. In our retrospective pilot study, we found that some of the proposed indicators vary both with the general quality of care and between out-patient clinics. In addition, some indicators were associated with the outcome of successful professional reintegration after stroke. Our data also allows an evaluation of the performance of the indicators in measuring process quality aims that show an association with long-term outcomes after stroke and are meaningful to patients and caregivers.

## Methods

### Instrument development

The working group was founded in August 2015 to identify a set of preliminary quality indicators. The process included a collection of suggestions and input from the group, followed by an estimation of the validity, reliability and relevance of the indicators as well as of their estimated variability with the quality of care. Next, a literature search for evidence linking process quality aims to improved functional outcomes was performed. After the systematic literature review, the published evidence was rated, and the indicators were further subjected to an external peer review. We adhered to processes suggested by recommendations of the “First Scientific Forum on Assessment of Quality of Care and Outcomes Research in Cardiovascular Disease and Stroke” of the American Heart Association and by the requirements for clinical performance measures for use in the German healthcare system [14, 15]. Estimation of evidence and effect size of the selected quality aims was based on an adapted concept used by Bakas et. Al. [16, 17] in a similar way.

Domains to be covered included core areas of diagnosis and treatment as recommended by guidelines (specifically diagnosis and training of memory, attention, executive function and emotional problems) as well as important interdisciplinary aspects of out-patient neuropsychological therapy such as cooperation with social workers (mediation of the patients into social care if needed).

The percentage of quality aims that were met always was given in reference to the total number of patients that a particular quality aim was applicable to. For example, the percentage of patients mediated into social care was given in reference to the total number of patients with an open need for social care. Mediation into social care hereby was defined as help with getting access to some form of social counseling. For some out-patient clinics, this meant setting up a meeting with a social worker, for others guiding the patient to help outside of the clinic. To make sure that the indicators really applied to the individual patients this was always documented in a separate question of the survey. For example, for indicators related to the therapy of executive function deficits, it had to be documented whether or not the patient indeed had a deficit in this area.

The definition of successful reintegration into work included all patients that could be reintegrated into some form of work, also if it differed from their job before the stroke as well as if it was a government-funded working program for persons with acquired brain injury.

For all indicators associated with a diagnosis of treatment of functional deficits the definition of correct testing and treatment was based on the appropriate guideline recommendations that were also handed to the therapists as a leaflet during retrospective data collection.

Further details on the quality indicators and their development can be found in the supplementary methods section, especially Supplementary tables 1, 2 and Table 1.

**Table 1:** Selection process of the indicators

Indicators (percent of patients having received appropriate diagnostics and treatment)	Rating	Include in pilot study (yes/no)	% quality aims met (n)	Range among centers
1. Documentation of peripheral or central deficits (*n* = 104)	7	Yes	80 (*n* = 83)	22–98
2. Standardized depression assessment (*n* = 104)^a^	9	Yes	69 (*n* = 72)	0–100
3. Indication for memory deficits (*n* = 85) and appropriate diagnostics^b^	8	Yes	96 (*n* = 82)	91–100
4. Suspected defects in attention and appropriate diagnostic^b^(*n* = 98)	8	Yes	98 (*n* = 96)	91–100
5. Suspected defects in executive function (*n* = 73) and appropriate diagnostics^b^	8	Yes	99 (*n* = 72)	91–100
6. Deficits in executive function (*n* = 73) and appropriate training^c^	7	Yes	71 (*n* = 52)	47–100
7. Attention deficits and appropriate training (*n* = 95)^d^	8	Yes	44 (*n* = 42)	0–88
8. Attention deficits and help to organize daily routines (*n* = 95)	7	Yes	87 (*n* = 83)	82–100
9. Severe memory problems and/or executive function deficits and inclusion of relatives in therapy (*n* = 64)	8	Yes	67 (*n* = 43)	47–100
10.offer to involve relatives (*n* = 104)	9	Yes	84 (*n* = 87)	44–100
11. Assessment of aims for participation in private and professional life (*n* = 104)	9	Yes	100 (*n* = 104)	
12.Open social-economic problems and mediation into provision of care (*n* = 62)	7	Yes	65 (*n* = 40)	5–100
13. Treatment plan for handling the emotional consequences (*n* = 104)	8	Yes	92 (*n* = 96)	80–100
14. Documentation that professional reintegration was successful (*n* = 72)^e^	10	Yes	63 (*n* = 45)	50–100
15. Counselling on fitness to drive (*n* = 104)	7	Yes	88 (*n* = 92)	60–96
16. Time between first contact in out-patient clinic and beginning of therapy < 1 month	10	Yes	63 (*n* = 66)	22–90

To decide on a set of indicators appropriate for further testing and external review, indicators were rated by the consensus group on a 10 point scale based on the strength of available evidence, expected effect size, estimated relevance for the patients and in a socio-economical context as well as in respect to reliability and validity of the indicators. Indicators were dropped if < 6 points were given. If two indicators covered the same area of neuropsychological aftercare the indicator with the higher score was selected. More details on the rating can be found in the supplementary methods section

^a^using standardized scores, ^b^according to guidelines, ^c^multiple training sessions including problem solving, managing aims, working under time pressure, self-management or meta-cognitive training (according to guidelines), ^d^attention-deficit specific training according to Sturm et al., ^e^out of patients wishing for professional reintegration

### Study design

The pilot study was prepared between January 2017 and November 2017 (Supplementary table 2). The study was designed as a retrospective cross-sectional sample, collecting real-life data from five out-patient clinics. Data were anonymized before final transfer to the study-center.

Generally, all patients that were at least 18 years of age and in neuropsychological therapy to recover from a prior stroke could be included. Data collection focussed on patients who started therapy in 2012 and onward because out-patient clinics operational in Germany since then could apply for a general license for reimbursement of post– rehabilitation out-patient neuropsychological therapy after stroke by the national health insurance. The duration of therapy after stroke on average was longer than 1 year and patient numbers treated per year were gradually building up after the said implementation of reimbursement.

A prerequisite for collecting complete information on therapy and its outcomes was for the patients to have finished the therapy. Therefore, most patients were included between mid-2013 to the beginning of 2015 and finished therapy in 2015–2016. Four of the five participating out-patient clinics ended up treating between 10 and 15 stroke patients per year. One clinic only occasionally applied for a case-specific license for out-patient treatment of stroke patients.

The therapists were asked to retrospectively screen their patient records starting 2012 and to work to include all stroke patients over 18 who had already finished therapy. Each clinic thereby was asked to collect data from at least 15 patients if possible.

However, to be able to get a representative picture for quality in diagnosis and treatment of the major neuropsychological deficits, three further instructions were given

1. Therapists were asked to include patients with attention, memory and executive function deficits in equal numbers (ending up with at least five in each group) if possible. When patients qualified for more than one of these diagnoses the group, they were allocated to was the group with fewer patients. When one group was already filled with five patients, therapists were encouraged to collect data for a group consisting of fewer than five cases if this still left enough patients to finally contribute a total number of 15 cases to the study.
2. The therapists were further instructed to collect data from patients that had an ischemic stroke and had already finished therapy. However, if out-patient clinics could only contribute fewer than 15 cases patients with a haemorrhagic stroke that had finished therapy, they could also be included (second priority).
3. Finally, if not enough patients were available from these two categories therapists could also include patients with ischemic or haemorrhagic stroke who had not yet finished therapy but for whom the frequency between two therapeutic sessions was more than 6 weeks apart. At this stage at the end of therapy, it was conceivable that therapeutic aims such as professional reintegration were either achieved or not achieved. All over, we aimed to include 75–110 patients from the five out-patient clinics and finally included a total of 104 patients all of whom had suffered from ischemic stroke. Supplementary figure 1 shows a flowchart of the study design with estimated numbers on exclusion because the minimum number of patients to be included had been (over)achieved or because patients had not yet finished therapy.

### Data collection and procedures

To ensure validity, questionnaires were designed targeting documentation of whether or not a certain therapeutic or diagnostic process had been performed. If the process was not documented it was considered to not have been done.

Before the start of data collection, the therapists received the questionnaire and an additional meeting was scheduled, to discuss open questions. After the first round of data entry by the therapist’s open questions and potential mistakes were discussed in individual meetings in the out-patient clinics, and mistakes were corrected where necessary.

### Statistics

Exploratory data analysis was performed with Stata version 14. Group differences between male and female participants were estimated. For the analysis of frequencies, the chi-square test was used. For continuous variables, normality was tested by the Shapiro Wilk test and was significantly rejected for all continuous variables except age. For the age at the beginning of therapy, we consequently used the T-Test for group comparisons. For the variables years in education, time since stroke, quality of care and Barthel index significance of differences was calculated by Mann-Whitney U Test. The reference category for effect estimates presented was male. Effect sizes are presented by Cohen’s d and odd ratios with 95% confidence intervals (CI) for ordinal and frequency variables respectively. Furthermore, a mixed logistic regression for the effects of quality of care on professional reintegration was calculated including the out-patient clinics as a random factor. The exposure variable was the overall quality of care or single quality indicators with low quality as the reference category. Further, fixed factors were included to control confounding. These were age (continuous variable), sex (categorical with male sex as reference), time spent in education (continuous variable) and need for counselling regarding socio-economic problems (categorical variable with, no need as the reference category). The out-patient clinic was included as a random factor.

Finally, not all patients included in our study had similar deficits over all cognitive domains. Therefore, deficit domain-specific indicators were not always applicable to all patients and the proportion of overall process quality at the individual level was always calculated as the percentage of the quality aims that applied to the patients (meaning that deficits in the indicator specific domain of diagnosis and treatment did exist in the individual patient’s case).

## Results

### Development of a preliminary set of quality indicators

**Fig. 1:**
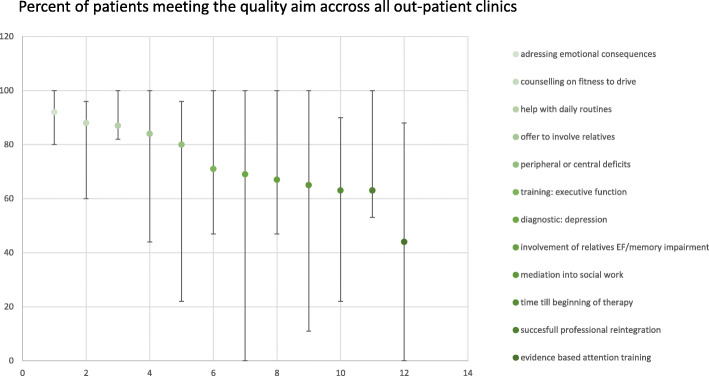
Percent of patients meeting the applicable quality aims across all out-patient clinics. Depicted are the 12 indicators showing stronger variability between the different out- patient clinics. Error bars mark the highest and the lowest percentage of quality met across the five out-patient clinics. EF = executive function

The development of the quality indicators and preparation of the retrospective pilot study took place during seven workshops of the working-group between August 2015 – November 2017 (Supplementary table 2). It included a definition of important areas of out-patient neuropsychological work, a collection of suggestions for relevant quality aims that could be used as indicators and a standardized literature search in the PubMed and Cochrane databases covering a total of 426 publications. Out of these, we used 22 publications, reviews and evidence-based guidelines (Supplementary table 1) as the major sources of supporting evidence during the selection process. Based on the evidence presented in this literature a first set of 21 potential indicators was specified. Further rating of relevance, evidence/effect size and validity/reliability of the measurements resulted in a reduced set of 16 indicators that finally were also rated for relevance and expected variability with care by two external experts (Table 1 and Supplementary table 3). In a next step the set of 16 indicators was tested in a retrospective pilot study. Overall percentages of patients meeting quality aims as well as the highest and lowest percentage across the five participating out-patient clinics are given in Fig. 1 and Table 1. Quality was especially high in the area of diagnosis and definition of treatment aims (Table 1). Here quality aims were met at a rate of nearly 100%. The twelve indicators that showed higher variability between patients (with > 5% of cases where the quality aim was not met) are depicted in Fig. 1. Process indicators with high variability of care included evidence-based attention training as described by Sturm et al. [18, 19] which was met in only 44%, of the cases (Fig. 1, Table 1). This was followed by the time between first contact and the beginning of treatment < 1 month (63% met), mediation into social work in case of need (65% met), the involvement of relatives for patients with executive function or severe memory deficits (67% met) and screening for depression (69% met) (Fig. 1, Table 1). The outcome of successful professional reintegration was met in 63% of the patients who had this treatment aim. A more detailed description of the literature references, indicators and their precise definition is also given in Supplementary table 1.

### Retrospective pilot study: dataset description and patient characteristics

Altogether 104 patients were included. Out of these patients in 90 (87%) memory deficits were suggested by location of acquired brain damage, prior clinical reports, reports of patients or relatives and/or assessment for memory deficits, 73 (70%) had deficits concerning executive function and 95 (91%) had attention problems. The five outpatient clinics contributed between 9 to 40 patients. The median overall duration of therapy was 1.25 years (min/max:0.25–3.5). Men were in the majority. To know whether this might have led to a systematic bias in other patient characteristics associated with gender in the next step, we also tested whether there were relevant gender differences concerning age, education, Barthel index, or the wish for professional reintegration (Table 2). Except that women were a bit younger at the beginning of therapy, we found no indication that there were major differences regarding these characteristics between men and women (Table 2). Barthel Index (BI) values, however, were only available for a subset (39%) of the patients. For those patients for whom data were available, index values suggested that almost none of the patients would have been classified as severely impaired when using standard scales to measure post-stroke impairment.

**Table 2:** Overview table: Overview of the patient population in the retrospective pilot study

	Women	Men	Total	*p*-value	Effect estimate Ref male^c^
N	40	64	104		
Time since stroke (month)^a^	5 (0–86)	6 (2–99)	6 (0–99)	0.15	−0.08(95%CI: - 0.45-0.29)
Age at beginning of therapy^a^	52 (30–83)	57 (33–81)	55 (30–83)	0.06	−0.38 (95% CI: − 0.77-0.02)
Years in education (*n* = 102)^a^	13 (8–20)	14 (8–20)	13 (8–20)	0.28	−0.2 (95%CI: − 0.61- 0.2)
% need for counselling in social law issues^b^	63% (*n* = 25)	58% (*n* = 37)	60% (*n* = 62)	0.63	1.22 (95%CI: 0.5–2.99)
% wish for professional reintegration^b^	73% (*n* = 29)	72% (*n* = 46)	72% (*n* = 75)	1	1.03 (95%CI: 0.39–2.79)
% of patients with standardized screening for depression	88 (*n* = 35)	58 (*n* = 37)	69% (*n* = 72)	< 0.01	5.11 (95% CI: 1.66–18.62)
Barthel-Index (*N* = 41)^a^	100 (95–100)	100 (50–100)	100 (50–100)	0.54	−0.06 (95%CI: −0.81-0.69)
% of overall quality of care^b^	87 (57–100)	79 (42–100)	83 (42–100)	< 0.01	0.6 (95% CI:0.21–1)

Except for age, no significant differences between men and women were seen. *P*-values represent results of chi-square or Man Whitney-U test, where appropriate. ^a^median (lowest/highest), ^b^% (n), ^c^Cohen’s d and (95% CI) for continuous variables. Effect estimates represent Cohen’s d for continuous variables and odds ratios for frequencies

Despite this, interesting differences in process quality of care between men and women were seen for overall quality (which was significantly higher in woman) and for the indicator 2 relating to screening for depression by the use of a standardized score. So the quality aim of screening all patients was met significantly more (chi-square *p*-value = 0.001) when the patients were female (Supplementary figure 2, Table 2).

### Association of overall process quality and single quality aims with the outcome of professional reintegration

Overall process quality per patient was measured as the percentage of the individually applicable quality aims met. The median overall rate was 83% and the mean rate was 81%. A reference area of 78% was defined by the lower 95% CI of the mean (Table 3). Patients with overall quality met at a lower rate than 78% were classified as receiving a lower overall quality of care.

**Table 3:** Overall performance and performance of the five outpatient clinics

Clinic	Median overall quality per patient in percent
A (min-max)	92 (69–100)
B (min-max)	70 (42–80)
C (min-max)	67 (50–78)
D (min-max)	92 (60–100)
E (min-max)	85 (67–93)
Overall (min-max)	83 (42–100)
	**Mean overall quality per patient in percent**
Overall (95%CI)	81 (95% CI: 78–83%, quality at or above reference when > 78%)

Overall performance and performance of the five outpatient clinics according to the defined quality indicators. Results represent median and mean values of overall quality met per patient in percent (*n* = 104)

**Fig. 2:**
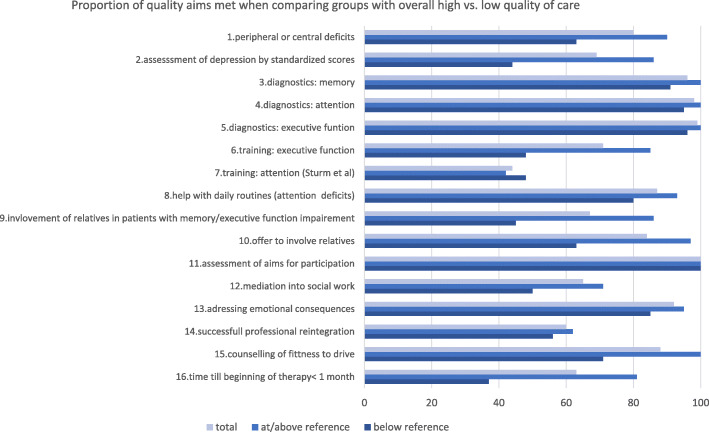
Proportion of process quality aims met when comparing groups with overall high vs. low individual quality of care. Shown is the overall rate of meeting the maximum of 15 process quality aims that could apply to individual patients. Depicted is the rate once for all patients, and once separated by the groups of patients from whom overall rate of meeting the process quality aims was above or below the reference area (defined as below the lower 95% CI of the mean rate of process quality met). Certain quality aims such as assessment of aims for participation were always met, while others showed a greater variation with overall process quality such as assessment of depression by standardized scores. Some indicators applied to all patients (for example assessment of depression by a standardized score), others only to subgroups with certain deficits or needs (for example training for executive function was applicable only to patients with executive function deficits or help with socio-economic problems was only applicable to those expressing a need in this area). Details on the definition of indicators can also be found in Supplementary table 1

Meeting the indicators related to screening for depression, time until the beginning of treatment and integration of the relatives into the therapy showed strong group differences with overall high or low quality of care. The fact that these indicators were met at higher rates in the group of patients who were also showing a generally higher level of process quality may suggest these indicators to be the drivers of the overall differences in quality of care (Fig. 2). In contrast, the indicators of evidence-based attention training - even though met in very few patients - did not differ with the overall quality of care and should not contribute to the separation of the groups with an overall higher or lower overall quality of care (Fig. 2). For the 72% of the patients with the treatment aim of professional integration, we calculated the effects that the overall meeting of process quality aims at or below reference had on the desired outcome of successful professional reintegration by mixed logistic regression. Results were corrected for age, sex, time spent in education and need for counselling regarding socio-economic problems as fixed factors and the out-patient clinic as a random factor. The odds for professional reintegration were significantly higher in patients with a process quality at or above reference (odds = 4.13 (95% CI: 1.17–14.67), *p*-value = 0.03). Furthermore, the need for mediation into social care also was significantly associated with a lower chance of professional reintegration (odds = 0.16 (95% CI: 0.04–0.63), *p*-value = 0.009). For the other covariates, no significant associations were observed. However, when classifying age as a categorical variable a reduced rate of professional reintegration in patients over 60 was observable (Supplementary figure 3).

Next, we tested single indicators that varied with the overall quality of care and were applicable to all patients in neuropsychological therapy for their sole effects on professional reintegration. These indicators were: the offer to involve relatives in the therapeutic process, the time from first contact until the beginning of treatment, the systematic screening for depression, clarification on whether the deficits of the patients were indeed were caused by central (and not peripheral) damage and the counselling on the ability to drive. Relevant effects were only observed for the offer to involve the patients relatives in the therapeutic process (odds ratio = 8.85 (95% CI = 1.3–60.16), *p*-value = 0.03), Supplementary table 4). Despite being the indicator with the lowest percentage of quality met (40%) having done an evidence-based attention training according to guidelines in our study showed no association with the outcome of professional reintegration (*p*-value = 0.85).

## Discussion

Stroke is a major contributor to disability and the second leading cause of death worldwide [1]. Furthermore, an estimated 80% of all stroke survivors experience some form of cognitive impairment during stroke recovery which can make regaining social and professional functioning after stroke difficult [2]. These numbers outline the potential relevance of neuropsychological therapy in correctly diagnosing and treating these patients for their cognitive deficits. However, especially in the out-patients setting the quality of neuropsychological therapy and its association with long-term outcomes remains unknown.

In this study, we aimed to develop a set of quality indicators that would cover major aspects relevant to out-patient neuropsychological diagnostics, treatment and care and reflect variations in the quality of out-patient neuropsychological therapy in a retrospective pilot study. The relevance of potential variation in care measured by the developed process indicators further was further evaluated by their association with important outcome quality aims (here professional reintegration). The developed indicators displayed different degrees of variability between patients and out-patient clinics leaving 12 indicators as potential candidates for further characterization in a prospective study (Table 1, Fig. 1). Furthermore, in our retrospective study higher overall process quality showed a trend for a positive association with the outcome aim of successful professional reintegration. Finally, when looking at single process indicators, namely the offer to involve the patients’ relatives into the therapeutic process, positively affected professional reintegration (Supplementary table 4).

### Selection of indicators

Indicators were selected to cover all relevant areas of neuropsychological aftercare after stroke.

Most of the developed indicators are process indicators. Measuring process indicators rather than outcome indicators can be beneficial, as outcome indicators more often are highly influenced by patient characteristics such as level of education, age and sex. This was also seen in our study, where professional reintegration seemed to be difficult in patients over 60 which is in good agreement with previous data showing older age to be a risk factor for successful professional integration and general participation [20, 21].

Confounders such as age, sex, education or the functional status of the patients at the beginning of therapy, however, often are not measured completely or may not be measured precisely enough, resulting in biased results and a relevant over - or underestimation of the association of the outcome indicators with quality of care. Process indicators in contrast can be measured directly as the percentage of patients for whom a specific process is fulfilled or not. Thereby they also sensitively reflect changes in care (meaning an improvement or worsening of keeping the recommended therapeutic processes) and correction for confounders often may not be necessary. Still, maintaining high process quality and keeping to a particular therapeutic process in question may not only depend on awareness for and monitoring of process quality in out-patient clinics but can be influenced by patients’ characteristics as well as by preconceptions of the therapists. For example, a high degree of physical disability may make meeting some of the therapeutic process quality aims in the current settings of out-patient neuropsychological therapy difficult or even impossible. On the other hand, certain patient characteristics may also interact with preconceptions of the therapists. In our study, we saw significant differences between the sexes especially concerning whether or not depression had been tested by a standardized score. As previous studies confirm [22] men generally report feelings of depression to a lesser degree than women after stroke. As a consequence, therapists might have regarded such a screening in men to be of less relevance.

### Variability with care

Due to the low general variability with care and between out-patient clinics, quality indicators related to keeping to standard diagnostic procedures or the definition of treatment aims may not represent ideal candidates for monitoring process quality. In contrast to this, indicators such as the mediation into care by social workers showed much more variability in general and between out-patient clinics. Incorporating such e innovative concepts may help to further improve neuropsychological therapy beyond the scope outlined in current guidelines and also to generate new evidence on tools that may be useful or necessary to obtain optimal treatment outcomes for the patients.

### Association of single process indicators with the outcome of professional reintegration

In our study, we observed an especially high association between the involvement of relatives in the therapeutic process and the outcome of professional reintegration.

Data from previous literature suggests that family involvement is important for long-term treatment success also in other medical conditions that may require changes in daily living that affect the relatives and caregivers as well as the patients [23]. However, in our retrospective study, we did not assess in detail whether the decision of neuropsychologists to offer involvement of relatives was influenced by prior knowledge about the patient, the family status, or on the willingness and capability of potential relatives to be involved. Consequently, the effects may result from a mixture of factors including a problematic family situation where involvement was simply not possible in combination with effects when family involvement was not offered for other reasons. In future prospective studies, additional data regarding family status and relations should be collected to understand the results in more detail. Despite this and irrespective of the exact circumstances the data suggests that the quality aim is important to obtain positive treatment outcomes and is a promising marker to monitor process quality in out-patient neuropsychological therapy.

The high rate of patients that did not receive evidence-based attention training as recommended by guidelines could be an indication that the training as is currently recommended [24] and used in clinical trials to study the effects of such training [25] may not be well suited for out-patient neuropsychological therapy. An explanation could be that the high frequency of training needed in combination with the high licensing costs for using validated computer-based home training may not be practical for some patients in the out-patient setting. However, the fact that meeting this quality aim was not found to be associated with the outcome of professional reintegration and that it was infrequently met also in patients with a higher general rate of process quality could indicate that the out-patient therapists did find adequate alternatives to standard training in the out-patient setting. Also, prospective data on whether such training was recommended or which kind of other non-standard trainings (meaning training not currently recommended by guidelines) may have represented a more practical alternative may help to explain the effects in more detail.

Apart from quality indicators, also the need for mediation into social care was associated with a reduced rate of successful professional reintegration. Patients with a documented need for mediation into care may need of specific assistance with reintegration such as government-funded vocational retraining programs or models for part-time work. The fact that a need for mediation into social work was associated with a lower rate of professional integration therefore could therefore indicate shortcomings associated with such socio-economic support for successful reintegration. In future prospective studies, this aspect should be investigated in more detail.

### Limitations

A major limitation for the development of quality indicators is the currently low level of evidence for neuropsychological therapy and training, and for out-patient post-stroke rehabilitation in general and even more concerning the specific combination. Therefore, the evidence supporting our indicators is limited. Nevertheless, using the set of quality indicators in larger prospective studies may help in generating new evidence regarding the effectiveness of neuropsychological interventions and help to further develop this first set of indicators. Moreover, features specific to the German healthcare system of course affect the performance of the quality indicators and may limit their usefulness in other health care systems.

When interpreting the odds ratios in our retrospective dataset it is important to keep in mind that those may overvalue the relative risk because the outcome of successful professional reintegration in our dataset is common. In the next step data on the relative risks should be collected in larger prospective studies.

Furthermore, we had only limited documented information on the general functional status (severity of post-stroke impairment as measured by standard instruments) at the beginning of the therapy available. Due to the retrospective nature of the current study, this information as well could not be obtained. More specifically, we had very little data on the Barthel index at the beginning of therapy as a potentially important confounder. The degree of severity as measured by the Barthel Index might affect the degree to which the current indicators and quality aims could represent realistic and representative expectations and treatment aims for stroke survivors in general. For the patients where data on Barthel Index values after stroke were available, they indicated that those patients were –apart from their cognitive deficits- otherwise not very severely affected by the prior stroke at the beginning of neuropsychological therapy. The out-patient therapists confirmed that in their experience, the barriers to attending out-patient therapy for more frail patients or patients with more severe motor-impairements indeed were high. Currently, there are very few existing out-patient clinics in Berlin. Consequently, these clinics are often far away from the patient’s place of residence, making it particularly difficult for severely impaired patients to reach the clinics. Therefore, an underprovision of care especially in this group of highly vulnerable patients seems likely. The fact that this information was often lacking in discharge letters from previous acute and rehabilitation clinics might also indicate another quality problem related to the management of intersections from in-patient to out-patient care. This information in combination with the functional status after therapy needs to be collected in future studies in more detail.

Finally, our current data came from a very small retrospective dataset where the neuropsychologists provided their own data. Because this study was designed as a pilot study to provide the first evidence on whether the developed indicator set could be used to measure differences in the quality of care, we did not aim to include much more than 100 patients. However, low patient numbers may have limited our power to show significant effects especially for the exploratory analysis on the association of certain aspects of process quality on professional reintegration. To be certain that the collected data was valid, we asked for written documentation of a therapeutic process. This may have helped to avoid a recollection bias but may as well have led to an underestimation of the real frequency of cases where quality aims were met and bias the results of our statistical analysis towards unity.

## Conclusion

In conclusion, the definition of the set quality indicators introduced in the current study can be considered a first step to allow measuring the quality of out-patient neuropsychological work after stroke. The present set of 16 indicators includes 12 that show reasonable variability between individual patients and different out-patient clinics and therefore might be suitable to measure differences in-process quality of care. First data further suggests that keeping to the measured therapeutic processes may indeed have relevant effects on therapeutic outcomes. These first results need to be validated in larger prospective studies in which more detailed data on the patients’ socio-economic situation and disease severity should be collected.

## Supplementary Information

**Additional file 1.**

**Additional file 2.**

## Data Availability

The datasets generated and/or analysed during the current study are not publicly available to protect the anonymity of the participating clinics who might be identified especially by persons involved with the neuropsychological work in Berlin when complete information about the contributed patient number, patient characteristics, mode of reimbursement and quality aims achieved were available at the out-patient clinic level. The developed questionnaire has not been previously published elsewhere. An English language version can be found in the supplementary materials.

## Abbreviations

CI: Confidence interval
