# Supplementary material for: Quality measurement of out-patient neuropsychological therapy after stroke in Germany: definition of indicators and retrospective pilot study
Source: BMC Neurol. 2021 Feb 17;21:76. doi: 10.1186/s12883-021-02092-0 (PMC7888117; doi:10.1186/s12883-021-02092-0)
Supplement: Supplementary file 1 — Additional file 1. [file 12883_2021_2092_MOESM1_ESM.docx]

1. Out-patient clinic
2. Sex: male/female
3. Age at the beginning of therapy: (years)
4. How many weeks passed since the stroke (weeks)
5. How many years did you spend in education (including school, professional or university education)?
6. Indicator 1: Percentage of patients with documented and guideline conform clarification on whether deficits are peripheral or central (applicable to all patients after stroke).

Is there documentation of clarification on whether deficits are peripheral or central available? (yes/no)

1. Indicator 2: Percentage of patients with documentation on whether depression was assessed by standardized scores (applicable to all patients after stroke)

Is there documentation on assessment of depression by a standardized score available? (yes/no)

1. Indicator 3: Percentage of patients with suspected defects in memory and documentation of diagnostic procedures according to guidelines

Is documentation with regard to suspected memory deficits available? (yes/no)

Is the patient positive for suspected memory deficits (for example lesion of relevant brain regions, report of relevant memory problems in daily life, report/observation of clinical signs for memory deficits) (yes/no)

If yes: name reason for suspected memory deficit

If yes: Is there documentation on execution of diagnostic procedures in accordance with guideline recommendations including the testing of additional cognitive performance indicators available?

If yes: Please name the diagnostic tests performed

1. Indicator 4: Percentage of patients with suspected defects in executive function and documentation of diagnostic according to guidelines

Is there documentation with regard to suspected executive function deficits available (yes/no)

Is the patient positive for executive function deficits (for example lesion of relevant brain regions, disregard for instructions, disorganized behavior) (yes/no)

If yes: name reason for suspected executive function deficit.

If yes: Is there documentation on execution of diagnostic procedures in accordance with guideline recommendations with at least one procedure for testing working memory, monitoring, cognitive flexibility, problem solving in combination with documentation on information and observations from relatives? (yes/no)

If yes: Please name the diagnostic tests performed

1. Indicator 5: Percentage of patients with suspected defects in attention and documentation of diagnostic according to guidelines

Is there documentation with regard to suspected attention deficits available (yes/no)

Is the patient positive for attention deficits (for example lesion of relevant brain regions, concentration deficits, inability to several things at the same time, sensibility towards noise observed or reported by previous therapists/medica doctors or relatives) (yes/no)

If yes: name reason for suspected attention deficit

If yes: Is there documentation of execution of diagnostic procedures in accordance with guideline recommendations with at least one procedure for attention intensity and selectivity? (yes/no)

If yes: Please name the diagnostic tests performed

1. Indicator 6: Percentage of patients with deficits in executive function and documentation of multiple training sessions including problem solving, managing aims, working under time pressure, self-management or meta-cognitive training

For patients with executive function deficits:

Is there documentation of multiple training sessions including problem solving, managing aims, working under time pressure, self-management or meta-cognitive training (yes/no)

If yes: please name the training performed

1. Indicator 7: Percentage of patients with attention deficits and documentation for attention deficit specific training according to Sturm et al.

For patients with attention deficits:

Is there documentation of multiple attention training sessions according to Sturm et al? (yes/no)

If yes please specify the training:

1. Indicator 8: Percentage of patients with attention deficits and help to organize daily routines

For patients with attention deficits:

Is there documentation of help to organize daily routines (yes/no)

If yes please specify:

1. Indicator 9: Percentage of patients with severe memory problems or executive function deficits for whom it was documented that relatives were included in the therapy

For patients with severe memory problems or problems with executive function:

Is there documentation for the inclusion of relatives into therapy? (yes/no)

1. Indicator 10: Percentage of patients with documented offer to involve relatives into the therapeutic process

Is there documentation of the offer to involve relatives into the therapeutic process? (yes/no)

1. Indicator 11: Percentage of patients with documentation regarding assessment of aims for participation in private and professional life (applicable to all patients after stroke)

Is there documentation of assessment of aims for participation in private and professional life? (yes/no)

1. Indicator 12: Percentage of patients with documentation of clarification of open need with social-economic problems and in case of need mediation into provision of care.

Is there documentation on clarification of open need with social-economic problems? (yes/no)

If yes: Was there open need? (yes/no)

If there was need. Was the patient mediated into care? (yes/no)

1. Indicator 13: Percentage of patients and relatives where there was a treatment scheme for handling the emotional consequences of the disease

Is there documentation on a developed treatment scheme for handling the emotional consequences of the disease? (yes/no)

1. Indicator 14: Percentage of patients with treatment aim of professional reintegration for whom such re-integration is successful

Is it documented that the patient had the treatment aim of professional reintegration (yes/no)

Is the therapy over (yes, therapy session>6 weeks apart, no)

If yes or therapy cycle >6 weeks apart: Was reintegration successful (yes/no)

1. Indicator 15: Percentage of patients with documented counselling on fitness to drive

counselling on fitness to drive. Is there documentation on counselling on fitness to drive (yes/no)

1. Indicator 16: time between first contact in out-patient clinic and beginning of therapy >1 month

Please indicate time after release from acute/rehabilitation hospital until first contact (month)

Please indicate time from first contact until beginning of therapy (month)

1. Is the therapy over? (yes, therapy session>6 weeks apart, no)

If yes when did therapy end (quarter, year)

Regular ending (check), early ending (check)

If therapy ended earlier than expected: patient dies (check), other reason (check)
