# Supplementary material for: Quality measurement of out-patient neuropsychological therapy after stroke in Germany: definition of indicators and retrospective pilot study
Source: BMC Neurol. 2021 Feb 17;21:76. doi: 10.1186/s12883-021-02092-0 (PMC7888117; doi:10.1186/s12883-021-02092-0)
Supplement: Supplementary file 2 — Additional file 2. [file 12883_2021_2092_MOESM2_ESM.docx]

**Supplementary Methods and Results**

**Supplementary methods**

Methodological requirements of quality and evaluation the quality of scientific evidence

Similar to a previous project ([1](#_ENREF_1)) the indicators were based on the best scientific evidence available. Measures should be valid and reliable. If necessary: case-mix adjustment of the participating institutions should be possible to avoid bias due to potential systematic differences in patient characteristics in the different out-patient clinics.

Furthermore, measurements must be possible without high effort and indicators must be sensitive to changes in the provision of care. Outcomes measured should be relevant to the patient’s quality of life and functionality and also with regard health economic considerations.

Constitution of consensus group and methodological approach of QI development

The consensus group med seven times between mid 2015 and end of 2017 accompanying all stages of QI-development, evaluation and preparation of retrospective data acquisition. It was constituted by the four out-patient therapist in Berlin and Potsdam who at that time had the general licences to get the therapy refunded by public health insurance as well as an out-patient clinic which applied at the health insurances for individual funding or treated patients who paid their therapy privately. Four of five neuropsychologist also had additional training as psychotherapists.

In the first two meetings the Donebian concept of quality control in the areas of structure, process and quality outcomes was introduced and the main areas and the work-flow of out-patient neuropsychological work to be covered by the indicators were defined and include:

1. Treatment and diagnosis of deficits in memory, attention, executive function and emotional consequences of the disease.

2. Defining treatment aims together with the patients,

3. Clarification on social-economic situation/open need,

4. Integration of relatives into the therapy and

5.Integration and documentation of diagnosis of accompanying medical/peripheral neurological problems.

During the following meetings suggestion for indicators were collected, criteria to define relevance and evidence of the indicators were aligned and a preliminary set of indicators was selected for further evaluation based on evidence, relevance, validity and reliability. Next, a final set of indicators was selected for further review by two external experts.

Literature search and specification of the indicators

For each suggested indicator standardized search algorithms in Pubmed and in the Cochrane database of systematic reviews were done. Furthermore, current evidence-based treatment guidelines covering stroke aftercare were screened for recommendations.

Details on definition of Validity and reliability evidence and effect size of the indicators

As for neuropsychological therapy the current published evidence is limited and large randomized trials have not been done extensively the concept by Bakas (2) (Bakas T, 2014), was simplified a bit.

Ranking of supporting evidence was based on a scale of 0-2 and equalled 2 when there was at least one randomised controlled trial in support of it and no conflicting results were published. Alternatively, evidence also equalled 2 if the existing literature had been evaluated by a systematic review and results supported effectiveness of the intervention, if it was recommended by evidence-based guidelines and/or if the advantage of the treatment aim was obvious without need for a study. The latter would for example be the case for reaching the therapeutic aim of professional re-integration.

Evidence level was defined as 1 when results of the published studies where partially contradictory or if there were only non-randomized studies, best practice guidelines or best practice recommendations available supporting it. Evidence level 0 was given when the indicator was only supported by the opinions of working group members or external experts. These indicators were not included further in the selection process.

Effect size also was graded on a scale from 0-2, depending on whether mainly negative effects had been published, whether positive and negative effects had been observed or whether the advantages were clearly predominant.

Relevance was defined to vary on a scale of 1-3. Categories were defined as follows: 1=low or medium impact on personal and/or professional life or well-being, mortality, recovery, participation **or** socio-economic impact; 2= moderate to high impact personal **and/or** socio-economic impact; 3= high personal and social/economic impact.

Validity/reliability could vary on an overall scale from 1-3. Reliability of the indicators was ensured by asking for proper documentation on the processes or outcomes in question. For example, with regard to “screening for depression” it was asked for whether there for documentation of assessment for depression by using a standardized score. Reliability varied between 0 and 1 and was rated 1 point when the indicator could be measured very exactly on a numerical scale (for example time till treatment). Validity of the indicators meaning how well the indicator could measure differences in the quality of neuropsychological aftercare varied between 0 and 2 and was defined by how much it was expected to vary with differences in quality of care between the clinics (1 point) and by how well the measured feature was estimated to represented the core work neuropsychological aftercare (1 point). Some of the indicators that were expected to show greater variability between the clinics at the same time however might not be as much part of what could be considered as the core area of neuropsychological work. Therefore, indicators covering either the one or the other of the two aspects often were selected.

If two indicators covered the same area of neuropsychological therapy, the indicator that reached a higher overall score was selected.

External review

The aspects of validity and representability, were also rated by two independent experts and their combined recommendations were taken into account (Supplementary table 2). The experts were asked to rate the indicators for how relevantly they estimate that the indicators represent the overall quality of important aspects of out-patient neuropsychological work and how well they might differentiate between the clinics with regard to quality of care. For each of these two aspects 0-2 points could be given, so that each of the two experts could give a maximum of 4 points per indicator and each indicator could get a maximum of eight points in total. If an indicator got at least 3 points in total from one of the two experts it was kept in the final selection.

Including the combined feedback from the two external experts confirmed the use of the set of 16 quality indicators in the retrospective pilot study.

**Supplementary figure 1**

**
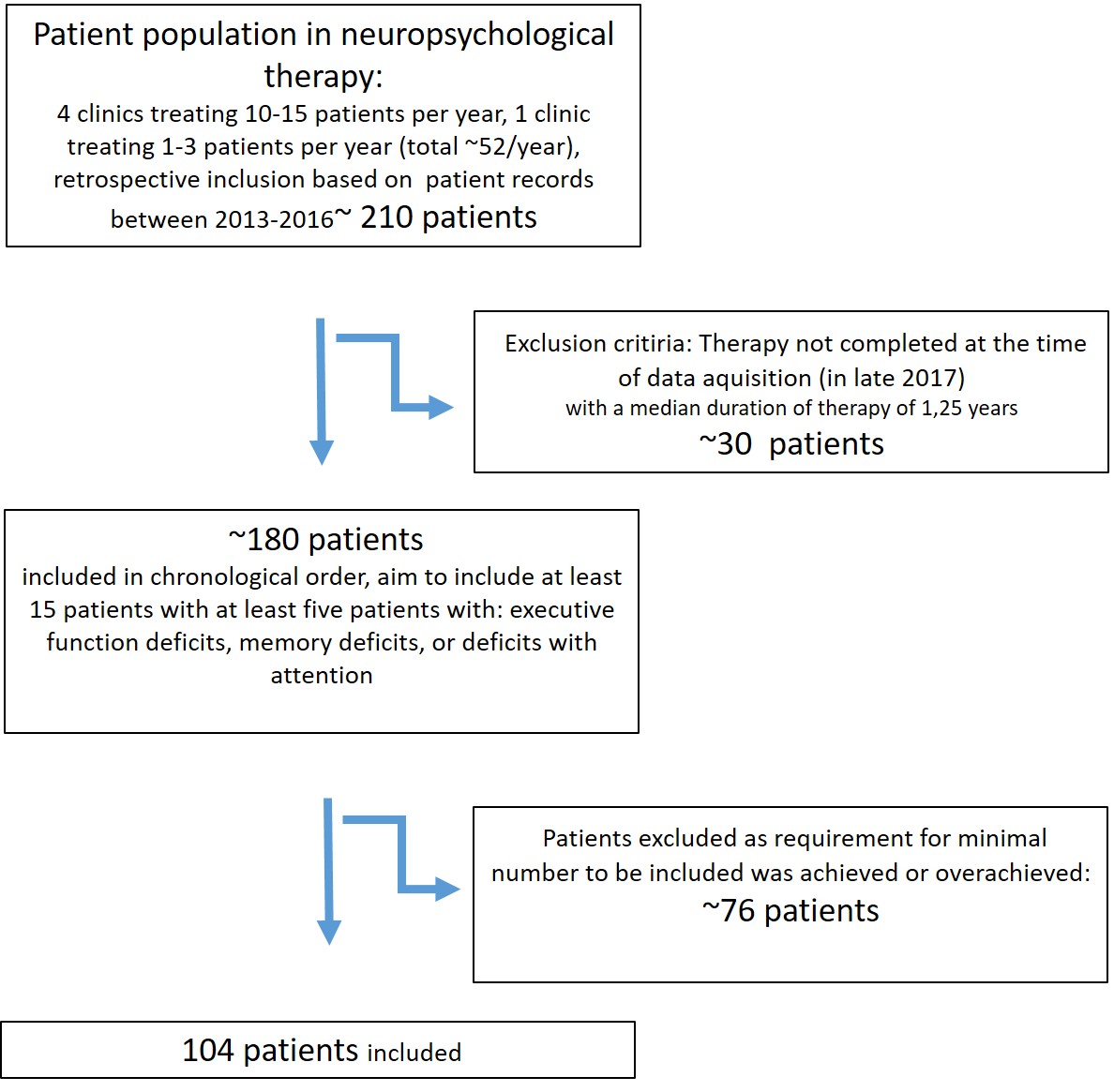
**

Supplementary Figure 1: Flowchart of inclusion of patients into the current dataset: Note: This study represents a retrospective analysis of real-life data collected in out-patient clinics and not a prospective inclusion of patients. Out-patient practitioners were instructed to chronologically browse through their previous patient’s records including patients as outlined above and in the material and methods section. Numbers given above represent estimates based on instructions for inclusion given, duration of therapy and estimates on the average of number of patients after stroke treated per year in each of the participating clinics. Included patients consisted of 61 cases with suspected memory, attention and executive function deficits, 5 with suspected memory and executive function deficits, 21 with suspected memory and attention deficits, 6 with executive function and attention deficits as well as of 3 patients with only memory, 7 with only attention and 1 with only executive function deficits.

**Supplementary Figure 2:** Screening for Depression


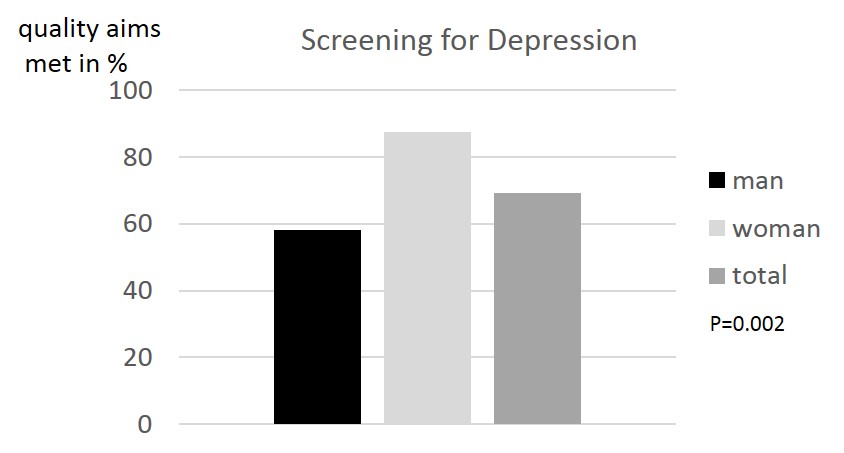


Supplementary Figure 2: Screening for depression was performed more frequently in man than in woman.

**Supplementary Figure 3**


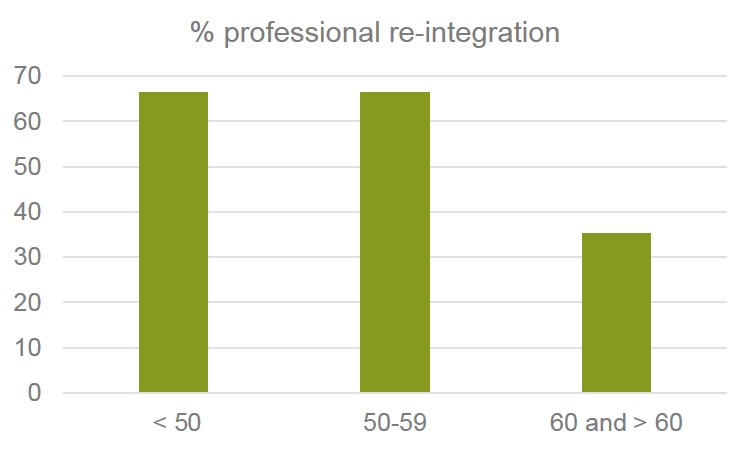


Supplementary figure 3: Percentage of professional re-integration in different age groups

**Supplementary table 1: Detailed description of indicators and expert score.**

Unless indicated otherwise indicators are applicable to all patients

|  | Collective expert score for representability and variability with care, maximum number of points=8 (4 by each expert) | **Reference** |
| --- | --- | --- |
| 1. Percentage of patients with documented and guideline conform clarification on whether deficits are peripheral or central (applicable to all patients after stroke). | 4 | GNP Guideline. Neuropsychologische Diagnostik und Therapie. Gesellschaft für Neuropsychologie (GNP). (Society for neuropsychological diagnostics and therapy, recommendations are based on best available evidence) |
| 2. Percentage of patients with documentation on whether depression was assessed by standardized scores (applicable to all patients after stroke) | 7 | Eskes GA, Lanctôt KL, Herrmann N, Lindsay P, Bayley M, Bouvier L, et al. Canadian Stroke Best Practice Recommendations: Mood, Cognition and Fatigue following Stroke Practice Guidelines, Update 2015. International Journal of Stroke. 2015;10(7):1130-40.  Grube MM, Dohle C, Djouchadar D, Rech P, Bienek K, Dietz-Fricke U, et al. Evidence-Based Quality Indicators for Stroke Rehabilitation. Stroke. 2012;43(1):142-6. |
| 3. Percentage of patients with suspected defects in memory and documentation of diagnostic procedures according to guidelines  This included documentation on whether or not there was reasonable cause to expect memory deficits due to a lesion of relevant brain structures, observation or report of relevant memory deficits in the daily routine or report or observation of clinical symptoms of memory deficits. For the patients for whom this was the case, it was further inquired whether documentation that guideline conform testing of different memory functions by standardized scores had taken place. | 6 | Arbeitsgemeindschaft wissenschaftlicher Fachgesellschaften (AWMF) S2e Leitlinie; Diagnostik und Therapie von Gedächtnisstörungen. Deutsche Gesellschaft für Neurologie (working group of scientific medical specialist societies, S2e Guideline on diagnostics and therapy of memory deficits, German Society of Neurology)  S2e classification indicates that recommendation are based on a systematic research of available evidence |
| 4. Percentage of patients with suspected defects in executive function and documentation of diagnostic according to guidelines  This included documentation on whether or not there was reasonable cause to expect executive function deficits due to a lesion of relevant brain structures, disregard for instruction or disorganized behavior and for the patients for whom this was the case documentation that guideline conform testing of function by standardized scores had taken place. | 6 | Arbeitsgemeindschaft wissenschaftlicher Fachgesellschaften (AWMF) S2e Leitline; Diagnostik und Therapie von exekutiven Dysfunktionen bei neurologischen Erkrankungen (working group of scientific medical specialist societies, S2e;  Guideline on executive dysfuction deficits in neurological illnesses.)  S2e classification indicates that recommendation are based on a systematic research of available evidence |
| 5. Percentage of patients with suspected defects in attention and documentation of diagnostic according to guidelines  This included documentation on whether or not there was reasonable cause to expect attention deficits due to a lesion of relevant brain structures, concentration deficits, intolerance against noise, inability to do several things at the same time and so forth. These signs may be reported by relatives or by previous therapists or may be observed in the course of therapy. For the patients for whom this was the case, there had to be documentation that guideline conform testing of function by standardized scores had taken place | 4 | Arbeitsgemeindschaft wissenschaftlicher Fachgesellschaften (AWMF) S2e Leitlinie; Diagnostik und Therapie von Aufmerksamkeitsstörungen bei neurologischen Erkrankungen  (working group of scientific medical specialist societies, Guideliens ;  diagnostics and therpay of attention deficits in neurological illnesses.)  S2e classification indicates that recommendation are based on a systematic research of available evidence |
| 6. Percentage of patients with deficits in executive function and documentation of multiple training sessions including problem solving, managing aims, working under time pressure, self-management or meta-cognitive training | 6 | Eskes GA, Lanctôt KL, Herrmann N, Lindsay P, Bayley M, Bouvier L, et al. Canadian Stroke Best Practice Recommendations: Mood, Cognition and Fatigue following Stroke Practice Guidelines, Update 2015. International Journal of Stroke. 2015;10(7):1130-40.  Cicerone KD, Langenbahn DM, Braden C, Malec JF, Kalmar K, Fraas M, et al. Evidence-Based Cognitive Rehabilitation: Updated Review of the Literature From 2003 Through 2008. Archives of Physical Medicine and Rehabilitation. 2011;92(4):519-30. |
| 7. Percentage of patients with attention deficits and documentation for attention deficit specific training. Including use of a validated training software and a frequency of training of 5 times/week and a minimum of 14 sessions according to Sturm et al. | 5 | Cicerone KD, Langenbahn DM, Braden C, Malec JF, Kalmar K, Fraas M, et al. Evidence-Based Cognitive Rehabilitation: Updated Review of the Literature From 2003 Through 2008. Archives of Physical Medicine and Rehabilitation. 2011;92(4):519-30.  Strurm W. Lehrbuch der Klinischen Neuropsychologie. (Textbook of clinical neuropsychology); Springer VS. 2009. |
| 8. Percentage of patients with attention deficits and help to organize daily routines | 6 | AWMF S2e Leitlinie; Diagnostik und Therapie von Aufmerksamkeitsstörungen bei neurologischen Erkrankungen  (working group of scientific medical specialist societies;  Guideline for  diagnostics and therpay of attention deficits in neurological illnesses.)  S2e classification indicates that recommendation are based on a systematic research of available evidence |
| 9. Percentage of patients with severe memory problems or executive function deficits for whom it was documented that relatives were included in the therapy | 5 | AWMF S2e Leitlinie ; Diagnostik und Therapie von Gedächtnisstörungen. Deutsche Gesellschaft für Neurologie  AWMF S2e Leitlinie; Diagnostik und Therapie von exekutiven Dysfunktionen bei neurologischen Erkrankungen  (working group of scientific medical specialist societies, Guideliens ;  diagnostics and therpay of executive dysfuction deficits and memory deficits in neurological illnesses.)  S2e classification indicates that recommendation are based on a systematic research of available evidence |
| 10. Percentage of patients with documented offer to involve relatives into the therapeutic process (applicable to all patients after stroke) | 7 | Eskes GA, Lanctôt KL, Herrmann N, Lindsay P, Bayley M, Bouvier L, et al. Canadian Stroke Best Practice Recommendations: Mood, Cognition and Fatigue following Stroke Practice Guidelines, Update 2015. International Journal of Stroke. 2015;10(7):1130-40. |
| 11. Percentage of patients with documentation regarding assessment of aims for participation in private and professional life (applicable to all patients after stroke) | 5 | Intercollegiate Stroke Working Party National clinical guideline for stroke. 4th edition. Royal College of Physicians. |
| 12. For patients with documented need for clarification of open social-economic problems: percentage of patients mediated into provision of care | 3 | T B. Evidence for Stroke Family Caregiver and Dyad Interventions. Stroke. 2014:2836-52.  Valtorta NK, Hanratty B. Socioeconomic variation in the financial consequences of ill health for older people with chronic diseases: A systematic review. Maturitas. 2013;74(4):313-33. |
| 13: Percentage of patients and relatives where there was a treatment scheme for handling the emotional consequences of the disease (applicable to all patients after stroke) | 8 | Eskes GA, Lanctôt KL, Herrmann N, Lindsay P, Bayley M, Bouvier L, et al. Canadian Stroke Best Practice Recommendations: Mood, Cognition and Fatigue following Stroke Practice Guidelines, Update 2015. International Journal of Stroke. 2015;10(7):1130-40. |
| 14. For all patients with the treatment aim of professional re-integration: percentage of patients for whom re-integration is successful | 4 |  |
| 15. Percentage of patients with documented counselling on fitness to drive (applicable to all patients after stroke) | 3 | Batool S, Roberts AP, Kalra L, Manawadu D. Health professionals’ knowledge of driving restrictions following stroke and TIA: experience from a hyperacute stroke centre. Postgraduate Medical Journal. 2014;90(1065):370-6.  Murie-Fernandez M, Iturralde S, Cenoz M, Casado M, Teasell R. Driving ability after a stroke: Evaluation and recovery. Neurología (English Edition). 2014;29(3):161-7. |
| 16. time between first contact in out-patient clinic and beginning of therapy >1 month (applicable to all patients after stroke) | 3 | Gemeinsamer Bundesausschuss, Psychotherapie-Richtlinie: Strukturreform der ambulanten Psychotherapie vom 16.06.2016,  psychotherapeutic guideline issued on 16.02.2016 by the committee of self- organization of health care providers, legislative and insurances in Germany |

Supplementary table 1: Literature supporting general evidence/effect size and for the given indicators presented. Furthermore, the indicators were rated by two independent experts. The experts were asked to rate the indicators for how representatively they regarded them for overall relevance and quality of neuropsychological work in stroke aftercare and by how well they might differentiate between the clinics. For each of these two aspects 0-2 points could be given, so that each of the two experts could give a maximum of 4 points per indicator and each indicator could get a maximum of eight points in total. If an indicator got at least 3 points in total from one of the two experts it was kept in the final selection.

**Supplementary table 2**

| August 2015 | Constitution of the working group |
| --- | --- |
| October 2015 | Definition of the areas of NP-Work |
|  | Definition of the methodological procedure (criteria for evaluation of literature and guidelines on potential quality aims) |
| October 2015-March 2016 | Collection of input on quality indicators (QI) from the group |
| March 2016- Mai 2016 | Definition of potential set of quality indicators covering the areas of work and literature search and evaluation of the QI regarding evidence and relevance |
|  |  |
| May 2016- July 2016 | Ranking of QI regarding evidence and relevance with the group |
| September 2016- January 2017 | Evaluation of the quality indicators by two independent experts and  Selection of a final set of 16 indicators for pilot testing |
|  |  |
| January 2017-November 2017 | Preparation of retrospective pilot study: Development of a case report form for the indicator set, definition of inclusion criteria for retrospective data collection and obtaining ethic approval for retrospective pilot study |
| November 2017-April 2018 | Data collection of the indicators in a retrospective pilot study |
| May 2018-September 2018 | Analysis of data from the pilot study |

**Supplementary Table 3:** process indicators not to be included in prospective study

| Indicator | rating | Reason not included prospectively |
| --- | --- | --- |
| 1. All patients with mild and moderate memory problems and with the aim to improve memory that receive a training for learning strategies to improve memory function at least 2-5 times a week. | 6 | Not included, dropped as variability was expected to be low and considering the milder form of impairment was relevant only for a subgroup of patients |
| 2. Percentage of patients with visual problems (for example visual neglect) and documentation of execution of a visual scanning training | 6 | Not included, dropped due to low score as this indicator was expected to be relevant only for a smaller proportion of the patients |
| 3. Availability of a computer assisted system for the detection of attention deficits in the out-patient clinic | 6 | Not included, dropped due to low score. Out-patient clinics emphasized every clinic would use similar systems. Consequently, there would have been hardly any variation between the clinics and the indicator did not seem suitable measure differences in the quality of care |
| 4. Availability of a computer assisted training system (cogni-plus) | 6 | Not included, dropped due to low score as out-patient clinics emphasized that if not available in the clinic it would be possible to get it licensed for home training of the therefore there would be no variation and low validity of the indicator to measure difference in treatment quality. Further the content here overlaps with indicator 7. |
| 5. Percentage of patients that aim to improve cognitive function and received standardized testing in the areas of attention, executive function and memory according to guidelines | 8 | Not included, dropped to enable domain specific indicators, otherwise duplication |

**Supplementary table 4**: Results of mixed logistic regression for association of single process indicators with the outcome of successful professional reintegration. Data were corrected for sex, age years of education, and need for mediation into social care as fixed factors and out-patients’ clinics as a random factor (n=70 subjects with documented aim of professional re-integration, documentation whether reintegration was successful and complete data on confounding factors). Tested were all process indicators that showed sufficient overall variability and that were applicable all and no just subgroups of the patients.

| Supplementary table 4 | Odds (95%QI) | p-value |
| --- | --- | --- |
| Offer to involve relatives (yes vs. no) | 8.85 (1.3-60.16) | 0.03 |
| Total time till beginning of treatment (change per year) | 1.30 (0.40-4.22) | 0.66 |
| Systematic diagnosis of depression (yes vs. no) | 0.82 (0.15-4.31) | 0.81 |
| Counselling on driving a car (yes vs. no) | 1.11 (0.09 -14.00) | 0.93 |
| Clarification if deficit central or peripheral (yes vs. no) | 0.49 (0.10-2.35) | 0.37 |

**References**

1. Grube MM, Dohle C, Djouchadar D, Rech P, Bienek K, Dietz-Fricke U, et al. Evidence-Based Quality Indicators for Stroke Rehabilitation. Stroke. 2012;43(1):142-6.

2. Bakas T, Evidence for Stroke Family Caregiver and Dyad Interventions. *Stroke***,** 2836-2852. Valtorta, N.K., and Hanratty, B. (2013). Socioeconomic variation in the financial consequences of ill health for older people with chronic diseases: A systematic review. *Maturitas* 74**,** 313-333
